# Level of health literacy and factors associated with it among school teachers in an education zone in Colombo, Sri Lanka

**DOI:** 10.1186/s12889-017-4543-x

**Published:** 2017-07-06

**Authors:** H. M. B. H. Denuwara, Nalika Sepali Gunawardena

**Affiliations:** 1 University of Colombo, Post Graduate Institute of Medicine, Colombo, Sri Lanka; 2 74/21C, Madawatta road, Weera mawatha, Depanama,, Pannipitiya, Sri Lanka; 3 National Professional Officer (Health Systems Analysis and Evidence), World Health Organization Country Office for Sri Lanka, 226, Bauddhaloka Mawatha, Colombo, 07 Sri Lanka; 4 540/3, Diyawanna Addara, Thalawatugoda, Sri Lanka

**Keywords:** Health promotion, HLS-EU, Self-reported competencies

## Abstract

**Background:**

Health literacy refers to people's competencies to access, understand, judge and apply health information in healthcare, disease prevention and health promotion. This study aimed to describe the level of health literacy and the factors associated with it among school teachers in an Education Zone in Colombo, Sri Lanka.

**Methods:**

A cross-sectional study among 520 teachers measured health literacy using the selfadministered, culturally adapted Sinhalese translation of Health Literacy Survey-European Union (HLS-EU). Health literacy assessment was based on self-reported competencies to access, understand, judge and apply health information in the domains of disease prevention, healthcare and health promotion. Based on a score, respondents were divided into four levels of health literacy as 'inadequate', 'problematic', 'sufficient' and 'excellent' as well as into two levels as 'limited' and 'adequate'. Factors associated with 'limited' health literacy was determined by using univariate analysis and assessing their associations using chi square test. Multivariate analysis was also done using multiple logistic regression to determine factors associated with limited health literacy controlled for confounding effects. A *p* value of 0.05 determined the significance.

**Results:**

The response rate was 96.5%. Mean age was 43years (*SD* = +9.75), 81.7% (*n* = 410) were females and 66.1% (*n* = 332) were graduates. Only 3.6% (*n* = 18) taught the subject health while 18.3% (*n* = 92) taught science. 'Limited' health literacy was found in 32.5% (95% CI 28.4%–36.6%) while 67.5% (95% CI 63.4%–71.6%), 61.2% (95% CI 56.9%-65.5%) and 6.4% (95% CI 4.3%–8.5%) showed 'adequate', 'sufficient' and 'excellent' levels, respectively. 'Problematic' and 'inadequate' health literacy were 31.5% (95% CI 27.4%-35.6%) and 1% (95% CI 0.1%–1.9%). Univariate analysis showed not being a member of health club/welfare group (*p* = 0.002), having not done any special course on health (*p* = 0.009), not getting an opportunity to participate/being exposed to a health awareness program (*p * = 0.007), visit to a medical practitioner/preventive health staff for six months (*p* = 0.049), not accessing print media to obtain general information (*p* = 0.007) and not accessing print media to obtain health information for six months (*p * = 0.009) and poor health knowledge (*p* = 0.036) to be factors associated with 'limited' health literacy that are modifiable. Nonmodifiable factors were age being ≤ 45 years (*p* = 0.025) and service as a teacher being ≤ 10 years (*p* = 0.012).

When multivariate analysis was performed, service as a teacher being ≤ 10 years (*p* = 0.042), monthly income ≤ Rs.50,000.00 (*p* = 0.024), not being a member of health club/welfare group (*p* = 0.034) and visit to a medical practitioner/preventive health staff for six months (*p* = 0.002), were found to be associated with limited health literacy among school teachers when adjusted to the effect of confounding of the other factors in the model.

**Conclusions:**

The high proportions of 'limited' health literacy among school teachers in the Colombo Education Zone and the need of interventional programmes should be brought to the attention of the policy makers in the education and health sectors. Improving health literacy among teachers and adoption of the Health Promoting School concept as a evidence based path to improve health literacy should be highlighted in the advocacy efforts. Identified factors associated with 'limited' health literacy should be taken into in the interventional efforts.

## Background

Health literacy refers to people’s “competencies to access, understand, judge, and apply health information in healthcare, disease prevention and health promotion” [1]. Being health literate facilitate healthy decision-making such as utilizing of health care services optimally, choosing healthy lifestyles or successfully dealing with the social determinants of health [2].

Current health systems place, more emphasis on self-care and consumers are expected to gain brand-new roles in finding facts, grasping the relevance and taking decisions related to health for themselves which requires improved health literacy [3]. Although health literacy was viewed initially as numerical skills and reading skills which are essential to operate adequately in the healthcare setting, health literacy concept has now developed further to include being competent in finding facts, analytical thinking, issues resolving, decision making and communication. It requires individual, social and cognitive skills that are essential to take part in the health system [1].

The school is a primary establishment in building the capital and health of nations [4]. There is a good relation in the concept of health promoting school (HPS) and health literacy [4]. The approach of HPS provides a plan that facilitate the achievement of all three levels of Nutbeam’s hierarchy of health literacy. It is undisputed that attainment of critical health literacy will be more easy if educational establishments adopt the health promoting school concept [4]. The concept of HPS guided the school health education in many nations. It is defined by offering students’ awareness in the lecture room regarding physical activity, diet, medication, dental health, safety, sexuality and affairs expecting that more facts would encourage building better attitudes on healthy conduct. To attain critical health literacy among students teachers to be health literate. Professional development opportunities are required to build up the ability of teachers to attain great levels of critical health literacy in themselves and in their learners [4].

The review of literature shows that studies assessing health literacy of teachers to be sparse. A cross-sectional study (*N* = 500) to assess health literacy and related factors in school teachers in Turkey in 2013 using the 6-item Newest Vital Sign tool revealed that 44.0% of the teachers had very limited, 29.8% limited and 26.2% adequate health literacy [5].

There is no ‘gold standard’ measure of health literacy [6]. Researchers have developed and used several tests to be used as proxy measures for this purpose. The latest of the comprehension tests of health literacy meant for the general population is the Health Literacy Survey- European Union (HLS-EU) developed and validated by the European Union. It is a 47 questions comprehensive, multidimensional tool which can be completed as computer-assisted or paper-assisted personal interviews [1].

Since its development in European Union, the HLS-EU has been used extensively to assess health literacy of general population groups. The health literacy survey in eight countries using the tool HLS-EU was conducted in Bulgaria, Austria, Greece, Germany, the Netherlands, Ireland, Spain and Poland (*n* = 8000, *n* = 000 per country) in the year 2011 and the results showed that as an average 47.6% of the population showed ‘limited’ health literacy [7]. Several Asian countries also have taken up health literacy surveys over the last few years, using culturally adapted and validated versions of HLS-EU. Japan [8], Taiwan [9], Kazakhstan [10], Malaysia [11], Indonesia [12], Philippines [13], Sweden [14] are these countries.

Some of the studies measuring health literacy have also assessed the factors associated with health literacy. Age [7–9, 12, 15–18], sex [7, 8, 12, 17–19], income [7, 9, 12, 16–18], abilities to pay for medication [11], level of education [4, 7, 9, 11–14], self-perceived social status [7, 9, 12, 15], watching health promoting television series [9, 15], knowledge [20], participating in community health programs [9, 15], receiving health-related training [9, 12] and frequency of visiting a medical doctor [7, 15, 17] were such factors.

Sri Lanka stands out as a country with high general literacy in the South East Asian region. In the year 2012 the general literacy rate was estimated as 95.7% for all population and 96.9% for males and 94.6% for females [21]. Health literacy among any population groups or factors associated with health literacy in Sri Lanka has not been assessed. Sri Lanka boasts for a well-established school health programme. Though the concept of health promotion through school settings has been principally accepted its implementation has much room to improve. Thus, school health programme was viewed as the intervention option for any gaps identified in the assessment of health literacy among school teachers. No previous attempts have been taken in Sri Lanka to validate any tool to assess heath literacy among general or specific population groups. In the absences, there is a backdrop of sparse empirical evidence on health literacy in any population groups in South East Asian region. The current study can be viewed as an effort to fulfill the gap.

## Methods

This was a descriptive cross-sectional study with an analytical component to identify factors. The study setting was the state schools of the Colombo Education Zone in the district of Colombo in the Western province, Sri Lanka.

The study population was the teachers of secondary school level or higher (grade 5 to advanced level). Sample size calculation was based on estimating the sample size for an unknown prevalence. Using a desired level of precision of 5% and a design effect of 1.2 and also allowing a 10% for non-response, the sample size was estimated as 520. Two stage cluster sampling method was the sampling technique used. A cluster was defined as a group of 40 teachers working in the same school. The Ministry of Education (MOE), classifies schools as Type 1AB, Type 1C and Type 2 based on the grades they teach and streams of study they offer. Allocation of the clusters to the different types of schools was based on probability proportionate to the size of the teacher population. The first stage of sampling was to select the required number of clusters relevant to each type of school. The second stage of sampling was to select the 40 school teachers from the school cluster and this was done by listing the eligible teachers in the selected school and selecting the required number of teachers randomly using the list as the sampling frame. The study was conducted during the period of June to October 2016.

Health literacy assessment was culturally adapted and Sinhalese translation of the self-administered version of the HLS-EU was done. As described earlier, the EU developed and validated HLS-EU to assess health literacy of general populations. It comprises 47 questions which measures health care health literacy, disease prevention health literacy and health promotion health literacy and general health literacy by requesting the respondents to rate their own competencies to access, understand, judge and apply health information. The competencies are rated in terms of difficulty/easiness with which they could perform what was described in each of the question in a Likert-type scale with 4 responses, scored from 1 (very difficult) to 4 (very easy).

In the process of adaptation of the HLS-EU, firstly the principle investigator and two competent persons fluent in Sinhala and English translated the questionnaire to Sinhala ensuring required cultural adaptations to the questions. Situations described and the examples in the HLS-EU were replaced by culturally appropriate local situations and examples that would be familiar to teachers. The translated version of the health literacy questionnaire was then presented to a group of stakeholders at a consultative meeting to assess its validity in terms of cultural suitability and appropriateness of the words. The panel of stakeholders comprised two Consultant Community Physicians, a researcher who had conducted research on health literacy in Sri Lanka, a teacher and a house wife. The items were modified till consensus of all the members in panel was obtained. Following confirmation of the consensual validity of the questionnaire, the panel was presented with the proposed scoring system by the HLS- EU and the cutoff values to classify the respondents to different categories of health literacy. The consensus of the panel was that the same scoring system and same cut offs are suitable to be used among the teachers in Sri Lankan setting. HLS (EU) comprises 47 questions and the responses are recorded according to a Likert-type scale with 4 responses, scored from 1 (very difficult) to 4 (very easy). The minimum possible test score is 47 while the maximum is 188 and then the scores are converted to percentages. The HLS-EU allows categorization of the study population into four as well as two levels of health literacy. When categorizing into four levels, health literacy test score of equal or below 50% is taken as ‘inadequate’ health literacy. The test score of above 50% and equal or below 66% is taken as ‘problematic’ health literacy. The test score of above 66% and equal or below 84% is taken as ‘sufficient’ health literacy. The test score of above 84% is taken as ‘excellent’ health literacy according to the method of the HLS-EU. In the categorization of two levels, the test scores 0% – 66% is taken as ‘limited health literacy’ and the scores of 67%–100% is taken as ‘adequate health literacy’.

Information on the potential factors associated with health literacy was through a set of closed ended multiple choice questions developed based on a conceptual framework that was derived from literature review.

The adapted HLS-EU and the questionnaire on the associated factors were incorporated into one and was pretested prior to use. The questionnaires were presented to the teachers following informed verbal consent and were requested to complete them then and there. For those who could not complete the questionnaire at that time were requested to place the completed questionnaires in the box that was provided for the purpose.

Ethical clearance was obtained from the Ethics Review Committee of the Faculty of Medicine, University of Colombo (EC-16-118). Data were entered and analyzed using SPSS version 21.

In assessing levels of health literacy, the study population was categorized into four categories of ‘inadequate’, ‘problematic’, ‘sufficient’ and ‘excellent’ and also into two levels as ‘limited’ and ‘adequate’ health literacy, as described above. The potential variables that were considered for the association of factors were cross tabulated with the two levels of health literacy and their associations were assessed by the chi square test using univariate analysis using SPSS 21 version. Multivariate analysis was also done using multiple logistic regression to determine factors associated with limited health literacy controlled for confounding effects. A *p* value of 0.05 was used to determine the significance.

## Results

The study included 502 participants with the response rate of 96.5% (502/520).

### Basic characteristics

Mean age of the study participants was 43 years (Standard Deviation (SD) = ± 9.75). Approximately half (48.0%, *n* = 241) of study population was in the age category 45 years – 60 years. A majority were married (89.0%, *n* = 447). Approximately half (54.9%, *n* = 276) of the study participants had 2–3 children.

Two thirds of the study participants (66.1%, *n* = 332) were graduate teachers. The study participants were inquired into the duration of the work experience as a teacher and the median was found to be 16.0 years (Inter Quartile Range (IQR) 6.5–26). Only 3.6% (*n* = 18) were teaching the subject health while 18.3% (*n* = 92) were teaching the subject science, at the time of the survey.

Of the study population 15.1% (*n* = 76) reported having hypertension, while 11.4% (*n* = 57) reported suffering from diabetes. Approximately three fourth (76.7%, *n* = 385) reported that at least one of their immediate family suffer from a non-communicable disease.

### Levels of health literacy

In assessing the four levels of health literacy, it was shown that only 6.4% (95% CI 4.3% - 8.5%) possess a level of health literacy of ‘excellent’. A majority of the study population belonged to the level of health literacy of ‘sufficient’ 61.2% (95% CI 56.9% - 65.5%). The proportions of ‘problematic’ and ‘inadequate’ levels of health literacy were 31.5% and 1% respectively.

In the assessment of health literacy in two levels, the results showed that a majority of the study population belonged to the level of health literacy of ‘adequate’ 67.5% (CI- 63.4%-71.6%). The proportion of the study population belonged to the level of health literacy of ‘limited’ was 32.5% (CI 28.4%-36.6%). Table 1 describes the proportions of two level health literacy in different domains.

**Table 1:** Distribution of the study population by the two level health literacy in different domains

Level of health literacy	Adequate health literacy		Limited health literacy	
	No	%	No	%
Disease Prevention health literacy (*n* = 502)	240	47.8	262	52.2
Health Care health literacy (*n* = 502)	356	70.9	146	29.1
Health Promotion health literacy (*n* = 502)	340	67.7	162	32.3
General health literacy (*n* = 502)	339	67.5	163	32.5

The proportion of ‘limited’ health literacy was highest for the domain of disease prevention health literacy (52.2%, *n* = 262). The proportion of adequate health literacy was highest for the domain of health care health literacy (70.9%, *n* = 356).

### Factors associated with ‘limited’ health literacy

Table 2 describes the association among basic factors and level of health literacy among the study population through univariate analyses.

**Table 2:** Distribution of the study population by level of health literacy and basic factors

Characteristics	Limited health literacy		Adequate health literacy		Total (*n* = 502)		Significance
	No.	%	No.	%	No.	%	
Age category							
Less than or equal to 45	101	36.7%	174	63.3%	275	100.0%	χ2 = 5 df = 1
Above 45^a^	62	27.3%	165	72.7%	227	100.0%	p = 0.025 OR = 1.6 (95% CI 1.1–2.3)
Period in the service as a teacher							
Less than or equal to 10 years	68	39.8%	103	60.2%	171	100.0%	χ2 = 6.3 df = 1
More than 10 years^a^	95	28.7%	236	71.3%	331	100.0%	p = 0.012 OR = 1.6 (95% CI 1.1–2.4)
Being a member of health club/welfare group in their community within last six months							
No	134	36.3%	235	63.7%	369	100.0%	χ2 = 9.4 df = 1
Yes^a^	29	21.8%	104	78.2%	133	100.0%	p = 0.002 OR = 2.1 (95% CI 1.3–3.2)
Done any special course in health related subjects within last six months							
No	156	34.2%	300	65.8%	456	100.0%	χ2 = 6.9 df = 1
Yes^a^	7	15.2%	39	84.8%	46	100.0%	p = 0.009 OR = 2.9 (95% CI 1.3–6.6)
Participated/exposed to any awareness program related to health within last six months							
No	111	37.1%	188	62.9%	299	100.0%	χ2 = 7.3 df = 1
Yes^a^	52	25.6%	151	74.4%	203	100.0%	p = 0.007 OR = 1.7 (95%CI 1.2–2.5)
Visited a medical practitioner or met with a member of the preventive health team to obtain medical treatment/ health advice during last six months							
Yes	121	35.3%	222	64.7%	343	100.0%	χ2 = 3.9 df = 1
No^a^	42	26.4%	117	73.6%	159	100.0%	p = 0.049 OR = 1.5 (95% CI 1.0–2.3)
Mass media mode accessed to obtain general information- Print media (newspapers, magazines)							
No	34	45.9%	40	54.1%	74	100.0%	χ2 = 7.4 df = 1
Yes^a^	126	29.9%	295	70.1%	421	100.0%	p = 0.007 OR = 2.0 (95% CI 1.2–3.3)
Mass media mode accessed to obtain health related information- Print media (newspapers, magazines)							
No	61	40.7%	89	59.3%	150	100.0%	χ^2^ = 6.8 df = 1
Yes^a^	99	28.7%	246	71.3%	345	100.0%	p = 0.009 OR = 1.7 (95% CI 1.1–2.5)
Level of knowledge on health							
Poor Knowledge	117	35.7%	211	64.3%	328	100.0%	χ2 = 4.4 df = 1
Good Knowledge^a^	46	26.4%	128	73.6%	174	100.0%	p = 0.036 OR = 1.5 (95% CI 1.0–2.3)

^a^Reference category

Being in the age category less than or equal to 45 years was significantly associated with ‘limited’ health literacy (*p* = 0.025). Being in a teacher for less than or equal to 10 years was significantly associated with ‘limited’ health literacy (*p* = 0.012).

Not being a member of health club/welfare group in their community within last six months was significantly associated with ‘limited’ health literacy (*p* = 0.002). Having not done any special course in health related subjects within last six months was significantly associated with ‘limited’ health literacy (*p* = 0.009). Not getting an opportunity to participate in an awareness program related to health/being exposed to any awareness program related to health within last six months was significantly associated with ‘limited’ health literacy (*p* = 0.007). Having visited a medical practitioner or met with a member of the preventive health team to obtain medical treatment/health advice during last six months was significantly associated with ‘limited’ health literacy (*p* = 0.049).

Having not accessed print media (newspapers/magazines) to obtain general information during last six months was significantly associated with ‘limited’ health literacy (*p* = 0.007).

Having not accessed print media to obtain health information during last six months was significantly associated with ‘limited’ health literacy (*p* = 0.009). Poor health knowledge was significantly associated with ‘limited’ health literacy (*p* = 0.036).

The associated factors identified to be significant in the multivariate analysis along with the adjusted odds ratios are depicted in Table 3.

**Table 3:** Significant factors associated with limited health literacy among school teachers

Factors	β	S.E.	Wald	df	Sig	Adjusted OR	95% CI for OR	
							Lower	Upper
Individual level factors								
1. Period in the service as a teacher ≤10 years	0.554	0.272	4.153	1	0.042	1.7	1.02	2.96
2. Monthly income ≤ Rs.50,000.00	0.569	0.252	5.090	1	0.024	1.8	1.07	2.89
3. Not being a member of health club/welfare group in their community within last six months	0.551	0.260	4.484	1	0.034	1.7	1.04	2.88
4. Visited a medical practitioner or met with a member of the preventive health team to obtain medical treatment/ health advice during last six months	0.722	0.237	9.258	1	0.002	2.1	1.29	3.27
Constant	−2.525	.867	8.478	1	.004	.080		

Period in the service as a teacher ≤10 years (adjusted OR = 1.7, 95% CI 1.02–2.96), monthly income ≤ Rs.50,000.00 (adjusted OR = 1.8, 95%CI 1.07–2.89), not being a member of health club/welfare group in their community within last six months (adjusted OR = 1.7, 95%CI 1.04–2.88) and having visited a medical practitioner or met with a member of the preventive health team to obtain medical treatment/health advice during last six months (adjusted OR = 2.1, 95%CI 1.29–3.27), were the factors associated with limited health literacy among school teachers when adjusted to the effect of confounding of the other factors in the model.

## Discussion

The results revealed that ‘limited’ health literacy was a considerable problem among school teachers in the Colombo Education Zone with approximately one third 32.5% (95%CI 28.4%- 36.6%) of the he study population in the level of health literacy of ‘limited’. There are no other local studies conducted to be compared to the health literacy among school teachers. The general literacy level in Colombo district as assessed in the population census was 94.7% and 95.3% for male and for females it was 94% [22]. The wide difference of the literacy proportions can be attributed to the complex mix of different skills needed in health literacy compared to general literacy.

Among the different domains of health literacy, the results of the present study showed that the school teachers were least literate in the domain of disease prevention (adequate health literacy- 47.8%) and were most literate in the domain of healthcare (adequate health literacy 70.9%). The proportions of study population with adequate health literacy in the domains of health promotion and general health literacy were 67.7% and 67.5%, respectively. Lowest health literacy being on disease prevention among an educated cross section of study population such as teachers highlights a worrying situation given the expectations of teachers being role models of the society and the agents to build health literacy skills among the students. On the other hand, this finding highlights the importance of emphasising the disease prevention aspects in health literacy building interventions.

The results related to health literacy of different domains of the present study was compared with other literature. Comparing the findings of 47.8% of ‘adequate’ health literacy in the domain of disease prevention among teachers in the present study with the other studies revealed that the cross-sectional study among Semarang (*n* = 1029) using HLS-EU showed the corresponding proportion to be similar (40.7%) [7]. So greater attention is needed to increase disease prevention health literacy among school teachers.

In the present study, the highest proportion of adequate health literacy was in the domain of health care health literacy (70.9%, *n* = 356). The cross-sectional study among Semarang people (*n* = 1029) using HLS-EU in Indonesian revealed that, adequate health care health literacy domain of respondents was 43.6% [7]. The higher proportion in the present study compared to the Indonesian study could be explained by the fact that the present study was on teachers who are more conversant with health care topics through their profession of teaching.

Comparing the findings of the present study on ‘adequate’ and ‘limited’ levels of health literacy with international studies assessing levels of health literacy, three studies revealed similar results while the others showed varying results.

A population survey of Taiwanese (*n* = 1493) adults of general population which was carried out in 2008 using Mandarin Health Literacy Scale revealed that 30.3% of adults to have low (inadequate or marginal) health literacy [23]. Although this finding was similar (32.5%) to the finding of ‘limited’ health literacy among the school teachers in the present study, difference in the study tool and the study population makes it meaningless to compare the two studies.

The other study with similar results is the research on functional health literacy in United States of America (*n* = 2659) among patients seeking care at two community hospitals. The results showed that one-third of patients who spoke English had marginal or inadequate health literacy [6]. Tool was not mentioned in the study. Again the differences of the study populations from the present study on teachers makes the comparisons irrelevant.

The finding of approximately one third (32.5%), of teachers with ‘limited’ health literacy in the present study is in keeping with the findings of the study among school teachers in Turkey in 2013 using the Newest Vital Sign tool which revealed that 29.8% to possess limited health literacy [5].

Among the studies which had revealed results different to the present study some have used the same tool, HLS-EU to measure health literacy as in the present study and others, different tools. Due to the difference in the study instruments and the study populations comparing these study findings is not possible.

The present study assessed the association of several factors with the level of ‘limited’ health literacy using univariate and multivariate analyses. Though being in the age category less than or equal to 45 years was significantly associated with ‘limited’ health literacy (*p* = 0.025) among school teachers of the Colombo Educational Zone the significance was not retained in the multivariate analysis confirming that significance was due to confounding (*p* = 0.646). Literature review revealed in two health literacy studies that old age was significantly associated with ‘limited’ health literacy [5, 8]. Other studies revealed that young age was significantly associated with ‘limited’ health literacy [7, 9, 15–17]. Further studies are required in different populations in Sri Lanka to explore the association between age and health literacy.

Period of the service as a teacher which was less than or equal to 10 years was significantly associated with ‘limited’ health literacy (adjusted OR = 1.7, 95% CI 1.02–2.96) in the multivariate analysis of the present study. In the absence of any other studies on health literacy among teachers there was no evidence to support or refuse this finding.

Not being a member of health club/welfare group in their community was significantly associated with ‘limited’ health literacy (adjusted OR = 1.7, 95%CI 1.04–2.88). This association can be explained by the fact that work of health club/welfare group in their communities are known to enhance the competencies of application of facts related to health in the domains of disease prevention and health promotion. This was further supported in other studies as well [11, 15].

Though not having undergone any special course in health related subjects (*p* = 0.009) and not getting an opportunity to participate in an awareness program related to health / being exposed to any awareness program related to health within last six months were significantly associated with ‘limited’ health literacy (*p* = 0.007) among the school teachers the significance was not retained in the multivariate analysis confirming that significance was due to confounding (*p* = 0.141, *p* = 0.062). These associations were revealed in several other studies [9, 12, 24].

Having visited a medical practitioner / any preventive health staff to obtain medical advice/treatment for them or anybody else during last six months was significantly associated with ‘limited health literacy’ among school teachers in the multivariate analysis of the present study (adjusted OR = 2.1, 95% CI 1.29–3.27). In the study among general population by the Health Literacy Survey, the ‘limited’ health literacy was seen in highest proportions among the persons with more than one long-term illness (61%), in those who reported six or more doctor visits within last 12 months (58.9%) and who reported a self-assessed health status of ‘very bad’ (78.1%) or ‘bad’ (71.8%) [4]. The nationwide survey conducted in Taiwan (aged >15 years, *n* = 2989) using the HLS-EU in 2013 revealed that higher general health literacy among men (β = −0.1, *p* < 0.05) as well as women (β = −0.1, *p* < 0.01) were negatively associated with frequency of visiting a medical doctor [15]. These findings confirm the theory that worse health demands for medical services most. Ideally one would expect that frequent contact with health personnel would improve the health literacy. The reverse shown may be attributed to the non-receipt and inadequate receipt of the relevant information and messages in these encounters.

Not having accessed print media to obtain general information and health information was significantly associated with ‘limited’ health literacy (*p* = 0.007, *p* = 0.009) in the univariate analysis. But these factors did not retain their significance in the multivariate analysis. The study in Turkey in 2013 using Newest Vital Sign tool revealed that adequate health literacy levels were significantly higher among teachers interested in healthy lifestyle topics in the media [5].

Having not accessed television to obtain general/health information was not significantly associated with ‘limited’ health literacy (general-*p* = 0.475; health- *p* = 0.088). In contrast to this finding other studies revealed that higher general health literacy was significantly associated with the higher frequency of watching health-related TV [9, 15].

Health knowledge is different to health literacy. Health literacy refers to people’s “competencies to access, understand, judge and apply health information in healthcare, disease prevention and health promotion” [1]. A cross-sectional survey conducted among 402 hypertensives and 114 diabetics in United States in 1994 to evaluate the relationship of patients’ knowledge of their chronic disease and Functional health literacy, which used Test of Functional Health Literacy in Adults (TOFHLA) to assess health literacy found that approximately half (48%) of the patients with diabetes or hypertension had inadequate functional health literacy and these patients had significantly less knowledge about their disease [20]. The point that diabetes patients with inadequate literacy had low knowledge scores in spite of taking part in formal education classes indicates that current educational strategies did not reach the huge number of patients with poor reading skills [20]. Although Poor Health knowledge was significantly associated with ‘limited’ health literacy (*p* = 0.036) the significance was not retained in the multivariate analysis confirming that significance was due to confounding (*p* = 0. 111).

Many studies revealed the association of low socioeconomic status and ‘limited’ health literacy [7, 9, 12, 16–18, 24]. Monthly household income equal to or less than 50,000 Rupees (*p* = 0.328) was not found to be significantly associated with ‘limited’ health literacy among the school teachers in the univariate analysis of the present study though was significantly associated with ‘limited’ health literacy in the Multivariate analysis (adjusted OR = 1.8, 95%CI 1.07–2.89) indicating that the univaruiate association was a result of the confounding effects.

This study, due to its inherent nature had to deal with some limitations. Although the findings of this study is externally valid for the Colombo Education Zone, extrapolation of the findings to other education zones of the country has limitations due to possible heterogenicity of characteristics of the school teachers in other geographical areas.

Health Literacy Survey – EU- Questionnaire (HLS-EU) used to assess the level of health literacy of the school teachers had not been previously validated in Sri Lanka. Though the version of HLS-EU used in the present study was adapted to be used among school teachers in the local study setting, only consensus validity which is a form of a judgmental validity was assessed. Reliability was also not assessed which is a limitation of this study. The scoring system and the cut off values used to classify the study population into different levels of health literacy were not based on a statistical form of validation but was based on the consensus of stakeholders. Use of internationally proposed scoring system and the cut off values may act as a limitation of the study affecting the internal validity of the results.

The temporal relationship between the identified factors associated with ‘limited’ health literacy cannot be elicited due to the cross sectional nature of the study.

## Conclusions

The results revealed that ‘limited’ health literacy was a considerable problem among school teachers in the Colombo Education Zone. Health literacy on disease prevention was the lowest among the school teachers. Some factors associated with ‘limited’ health literacy among school teachers in the Colombo Education Zone were modifiable.

The authorities of the Ministries of Education and Health should be advocated on the considerable problem of ‘limited’ health literacy among school teachers in the Colombo Education Zone and the need of corrective interventions. The Health Promoting School concept as an evidence based path to improve health literacy should be highlighted in the advocacy efforts. The fact that Sri Lanka’s school health programme is built on the principles of health promoting school concept and its revival would serve as the basis for the proposed interventions to improve health literacy among the teachers and thereby to build the critical health literacy among students should be emphasized in the advocacy efforts. It is recommended that the identified modifiable factors associated with ‘limited’ health literacy should be taken into consideration when designing the targeted interventional activities. Encouraging teachers to be members of health club/welfare group in their community is another policy level effort that can be recommended to improve health literacy. The identified non-modifiable factors should be used to identify the groups of teachers who should be given priority in interventional efforts.

The present study translated and culturally adapted the HLS-EU to the Sri Lankan setting facilitating future assessment of health literacy in the country.

## Abbreviations

95% CI: 95% confidence interval
DF: Degree of Freedom
HLS-EU: European health literacy survey
IQR: Inter Quartile Range
MHLS: Mandarin Health Literacy Scale
OR: Odds ratio
SD: Standard deviation
TOFHL: Test of Functional Health Literacy in Adults
